# Comparison of treatment methods of appendiceal mass and abscess: A prospective Cohort Study

**DOI:** 10.1016/j.amsu.2019.10.016

**Published:** 2019-10-24

**Authors:** Zaza Demetrashvili, George Kenchadze, Irakli Pipia, Kakhi Khutsishvili, David Loladze, Eka Ekaladze, Giorgi Merabishvili, George Kamkamidze

**Affiliations:** a Department of Surgery, Tbilisi State Medical University, 33 Vazha-Pshavelaave, 0177, Tbilisi, Georgia; b Department of Surgery, Kipshidze Central University Hospital, 29 Vazha-Pshavelaave, 0160, Tbilisi, Georgia; c Institute Medical Research, Ilia State University, 3/5 Cholokashviliave, 0162, Tbilisi, Georgia; d Department of Biochemistry, Tbilisi State Medical University, 33 Vazha-Pshavelaave, 0177, Tbilisi, Georgia; e Department of Immunology and Infectious Diseases, University of Georgia, 47 Tashkenti Str, Tbilisi, 0160, Georgia

**Keywords:** Acute appendicitis, Appendiceal mass, Appendiceal abscess, Appendectomy

## Abstract

**Background:**

The aim of our prospective study is to compare and analyze the results of two treatment methods of appendiceal mass and abscess: emergency surgery and conservative treatment with and without interval surgery.

**Materials and methods:**

74 Patients with the diagnosis of appendiceal mass or abscess were enrolled in this study. The patients were assigned into two groups: the emergency surgery group and the conservative management group. The conservative management group was subdivided into two groups: interval surgery group and the ambulatory follow-up observation group without interval surgery. Several clinical characteristics were determined and compared between the groups. Among patients who underwent surgery, the surgical methods, operation time, postoperative hospitalization period, and post-surgical complications were analyzed. In the ambulatory follow-up observation group, recurrence of appendicitis was assessed.

**Results:**

Comparison of the emergency surgery group and interval surgery group revealed that the interval surgery group was characterized by shorter operation time (P = 0.008), a smallernumber of postoperative complications (P = 0.02) and also shorter postoperative hospital stay (P = 0.009). In the ambulatory follow-up observation group, recurrence of appendicitis developed in 3 (13%) patients. US or CT-guided PCD was performed in all 3 patients on the conservative treatment stage. Comparing the interval surgery and recurrent appendicitis groups revealed statistically significant difference: operation time (P = 0.04) as well as postoperative hospital stay (P = 0.04) were shorter in recurrent appendicitis group. In 3 (4.1%) patients, the cause of the appendiceal mass was caecal cancer (2 cases) and Crohn's disease.

**Conclusion:**

Conservative treatment without interval surgery seems to be the preferred method for treatment of appendiceal mass and abscess. Patients can be operated on only in case of recurrence of appendicitis. US or CT PCD of appendiceal abscess presents the risk-factor for the development of recurrence of appendicitis. CT and colonoscopy within 4–6 weeks after completing the conservative treatment is recommended to be performed in all patients.

## Highlights

• Conservative treatment without interval surgery should be the method of choice for treatment of appendiceal mass and abscess.
• Patients should be operated only in case of recurrence of appendicitis.
• US or CT PCD of appendiceal abscess present the risk-factor for the development of recurrence of appendicitis.
• All patients should be performed CT and colonoscopy in 4–6 weeks after completing the conservative treatment.

## 1 Introduction

Acute appendicitis is one of the most frequent acute surgical diseases. The inflammation in acute appendicitis may sometimes be fixed by the patient's own defense mechanisms, such as, by the formation of an inflammatory mass (an appendiceal phlegmon) or a circumscribed abscess (an appendiceal abscess), often presenting as a palpable mass. This complication occurs in 2–7% of all cases of appendicitis [[1], [2], [3]].

Management of an appendiceal mass and abscess can either be performed as operative or conservative. More evidence is needed to clarify, which method is superior [1,2]. Immediate appendectomy may be technically demanding because of the distorted anatomy and difficulties in closing the appendiceal stump because of the inflamed tissues. Due to the above mentionedfactors, the operation could be finished with the colonic resections (ileocecectomy or right hemicolectomy) [[3], [4], [5]].

Conservative treatment with interval appendectomy traditionally remains as the gold standard of management. The need for interval appendectomy after a successful non-surgical treatment has recently been challenged as the risk of recurrence of appendicitis, because it is relatively rare [1,4,6]. After successful non-surgical treatment of an appendiceal mass, the true diagnosis is uncertain in some cases and underlying diagnosis of cancer or Crohn's disease (CD) could be delayed [3,4,7,8].

The aim of our prospective study is to compare and analyze the results of two methods of treatment of appendiceal mass and abscess: emergency surgery and conservative treatment with and without interval surgery.

## 2 Materials and methods

From January 2006 to August 2018, patients over 18 years of age, who were treated with diagnosis of appendiceal mass or abscess were enrolled in this prospective study. The patients were treated at the Surgery Department of our hospital. The patients were diagnosed by physical examination, ultrasound (US) examination and computed tomography (CT). The inclusion criteria were the diagnosis of appendiceal mass or abscess, and patient's consent to participate in the study. The exclusion criteria were patients' preference for either treatment method, patients' refusal to participate in the study, immunocompromised patients, and patients in the American Society of Anesthesiologists (ASA) groups 4 and 5. The patients were assigned to one of the following groups: those who have undergone emergency surgery - defined as the emergency surgery group (Group 1); those who were treated with conservative management, such as with antibiotics, with or without percutaneous drainage (PCD), guided by US or CT - defined as the conservative management group (Group 2). The Indication of percutaneous drainage was the existence of an appendiceal abscess. The conservative management group (Group 2) was subdivided in two groups: the interval surgery group, whose patients underwent surgery at a certain time after the time of initiation treatment (Group 2a), and the ambulatory follow-up observation group, whose patients underwent ambulatory follow-up observation without interval surgery (Group 2b). All surgical operations were performed by one team of three skilled general surgeons.

The clinical characteristics were collected for each patient: gender, age, major symptoms, duration of symptoms prior to admission, heart rate, body temperature at time of admission, leukocytes count, the presence or absence of a mass in the right iliac fossa, size of abscess and associated chronic diseases. Among patients who underwent surgery, the surgical methods, operation time, postoperative hospitalization period, and postsurgical complications were analyzed. In the ambulatory follow-up observation group (Group 2b), recurrence of appendicitis (Group 2c) during the ambulatory follow-up observation period were assessed. The follow-up observation period was from the day of the first visit to the most recent visit to outpatient clinic. The follow-up period for ambulatory follow-up observation group ranged from 7 to 67 months (49.7 ± 21.5 months).

The study was reviewed and approved by the Institutional review boards of the Tbilisi State Medical University (Tbilisi, Georgia) and Kipshidze Central University Hospital (Tbilisi, Georgia) ethics committee. The study was registered on researchregistry.com (UIN: 1765).

The research is reported in line with the Strengthening the Reporting of Cohort Studies in Surgery (STROCSS) criteria [9].

## 3 Statistical methods

Sample size calculation was performed for *t*-test to compare means of continuous variables for the following parameters: E/S = 0.5, Power = 80%, alpha = 0.05.

Descriptive statistics methods were used to characterize each variable. Comparison of continuous variables was performed by independent samples *t*-test or the Mann-Whitney *U* test according to the normality of the variables. Categorical variables were evaluated by two-tailed Chi-square test or Fisher's exact test where appropriate (for expected frequencies <5). The threshold for statistical significance was set to P < 0.05. The statistical tests were performed by IBM SPSS statistics package v23.0 (IBM Corporation, Armonk, New York).

## 4 Results

**Fig. 1:**
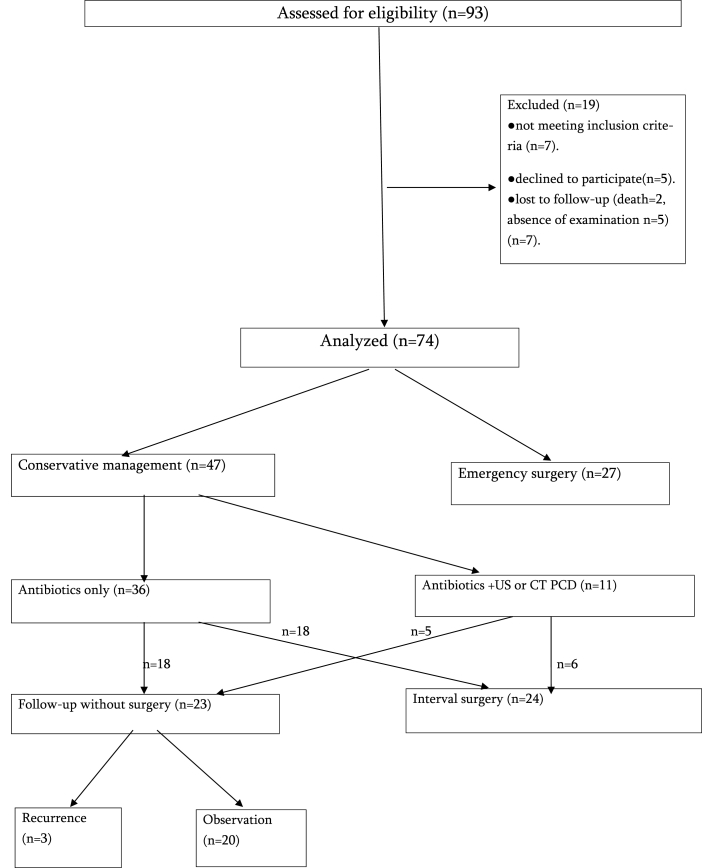
Study flow chart.

1587 patients with the diagnosis of acute appendicitis were treated during the study period. 93 (5.9%) patients had appendiceal mass and abscess. Among them, 19 patients have been excluded from the study. From these 19 patients, 7 patients have not met inclusion criteria, 5 patients declined to participate and information about 7 patients was lost during the time of observation (5 patients were not coming for examination, 2 patients died during observation period, the causes of death were all non-appendicitis related). Accordingly, 74 patient's data (27 patients from emergency surgery group and 47 patients from conservative management group) have been analyzed (Fig. 1). For diagnosis of Appendiceal mass and abscess, 74 patients received US, and 50 patients received CT testing. The age of the patient in the emergency surgery group ranged from age 18–79, and the age of patient's in the conservative management group ranged from 18 to 84.

The clinical characteristics of the emergency surgery and conservative management groups were not statistically different (Table 1).

**Table 1:** Comparison of clinical characteristics between the emergency surgery and the conservative management groups.

Characteristics	Group 1	Group 2	P value
	(n = 27)	(n = 47)	
Male:Female	16:11	23:24	0.47
Mean age (yr)	32.5(11.7)	35.2(12.4)	0.52
Duration of symptoms (day)	6.9(4.8)	7.7(5.6)	0.54
Pain	25(92.6)	42(89.7)	1
Nausea and vomiting	15(55.6)	23(48.9)	0.63
Mass	12(44.4)	25(53.2)	0.63
Body temperature (°C)	37.4(1.6)	37.1(2.0)	0.51
Heart rate (pulse/min)	92.7(28.6)	89.4(31.7)	0.66
WBC count (/mm³)	12.957(5637)	13.361(7212)	0.8
Size of abscess (cm)	3.9(2.1)	4.7(2.8)	0.2
Comorbidities			
Cardiovascular	5(18.5)	8(17.0)	1
Respiratory system	2(7.4)	2(4.2)	0.62
Diabetes	1(3.7)	2(4.3)	1

Group 1, emergency surgery group; Group 2, conservative management group; WBC, white blood cells.

Data are expressed as mean (SD) or absolute number of patients (%).

In the emergency surgery group (Group 1), an ileocecectomy and right hemicolectomy were performed in 7 cases (25.9%), due to severe inflammation and adhesion around the ileocecal region. Appendectomy was performed in the rest 20 cases. Histopathologic examination of the sectioned preparations confirmed perforated appendicitis in 26 patients (out of 27 patients) and caecal cancer – in one patient, who has been operated by right hemicolectomy. Postoperative complications developed in 6 patients (22.2%) (wound infection – in 4 cases, anastomotic leak and intraabdominal abscess – in 2 cases). None of 27 patients died.

Among patients of conservative management group (Group 2), 36 patients were treated only with antibiotics, and 11 patients were treated with antibiotics in parallel with US or CT-guided PCD. Interval surgery, after conservative treatment, was performed on 24 patients (Group 2a), and 23 patients underwent only out-patient follow-up observation, without interval surgery (Group 2b).

In the interval surgery group, appendectomy was performed in 22 cases, one ileocecectomy and one right hemicolectomy. An Ileocecectomy was performed in the patient who was under US-guided percutaneous drainage of appendiceal abscess. The patients' condition changed for the worse on the 9^th^day after drainage, patient was operated on the 12th day due to the progressed intraabdominal abscess. Histopathologic examination of the sectioned preparation confirmed the diagnosis of Crohn's disease. A right hemicolectomy was performed in the patient with intestinal fistula formed after the CT-guided percutaneous drainage of appendiceal abscess. This patient was diagnosed with caecal cancer by the colonoscopy, CT and right hemicolectomy was performed 5 weeks after initiation of the conservative treatment. All 22 appendectomies were performed 6–8 weeks after the conservative treatment. In all 22 cases, patients underwent CT and colonoscopy before surgery. No postoperative complications were observed. None of 24 patients died.

Comparison of the emergency surgery group and interval surgery group revealed that interval surgery group was characterized by shorter operation time (95% CI 8.39-52.82 P = 0.008), a smaller number of postoperative complications (95% CI 1.23-9.57 P = 0.02) and also by shorter postoperative hospital stay (95% CI 0.60-4.0 P = 0.009) (Table 2).

**Table 2:** Comparison of surgical outcomes between the emergency surgery and interval surgery groups.

	Group 1	Group 2a	P value
	(n = 27)	(n = 24	
Operations			
Appendectomy	20(74.1)	22(91.7)	0.15
ileocecectomy	5(18.5)	1(4.2)	0.2
right hemicolectomy	2(7.4)	1(4.2)	1
Operation time (min)	117.7(47.2)	87.1(28.1)	0.008

Postoperative complications	6(22.2)	0(0)	0.02

Postoperative hospital stay (day)	10.4(3.1)	8.1(2.9)	0.009


Group 1, emergency surgery group; Group 2a, interval surgery group.

Data are expressed as mean (SD) or absolute number of patients (%).

Clinical characteristics of the interval surgery group (Group 2a) and ambulatory follow-up observation group (Group 2b) were not statistically significantly different (Table 3).

**Table 3:** Comparison of clinical characteristics between the interval surgery and follow-up groups.

Characteristics	Group 2a	Group 2b	P value
	(n = 24)	(n = 23)	
Male:Female	10:14	13:10	0.39
Mean age (yr)	34.4(15.3)	31.7(14.2)	0.58
Duration of symptoms (day)	8.0(4.7)	7.1(3.9)	0.48
Pain	22(91.7)	20(87)	0.67
Nausea and vomiting	13(54.2)	10(43.5)	0.56
Mass	12(50)	13(56.5)	0.77
Body temperature (°C)	37.2(1.4)	36.9(1.2)	0.44
Heart rate (pulse/min)	87.7(26.4)	90.2(22.5)	0.73
WBC count (/mm³)	12.912(5882)	13.068(6031)	0.93
Size of abscess (cm)	5.1(2.1)	4.9(2.8)	0.78
PCD	6(25)	5(21.7)	1
Comorbidities			
Cardiovascular	5(20.8)	3(13.0)	0.7
Respiratory system	1(4.2)	1.(4.3)	1
Diabetes	1(4.2)	1(4.3)	1

Group 2a, interval surgery group; Group 2b, follow-up group; PCD, percutaneous drainage.

Data are exprexxed as mean (SD) or absolute number of patients (%).

In the ambulatory follow-up observation group (23 patients), CT and colonoscopy were performed on all patients 6–8 weeks after conservative treatment.

In ambulatory follow-up observation group, recurrence of appendicitis developed in 3 (13%) patients (Group 2c). In all 3 cases, recurrence developed in 3–4 months after completion of the conservative treatment. Worth to mention, that US or CT-guided PCD was performed in all 3 patients due to appendiceal abscess on the conservative treatment stage. Appendectomy was done in all these cases. No postoperative complications were developed. None of 3 patients died.

Comparing the interval surgery and recurrent appendicitis groups revealed statistically significant difference: operation time (95% CI 0.94-70.46 P = 0.04) as well as postoperative hospital stay (95% CI 0.25-7.35 P = 0.04) were shorter in recurrent appendicitis group (Table 4).

**Table 4:** Comparison of surgical outcomes between the interval surgery group and the surgery group for recurrence of appendicitis.

	Group 2a	Group 2c	P value
	(n = 24)	(n = 3)	
Operations			
Appendectomy	22(91.7)	3(100)	1
ileocecectomy	1(4.2)	0(0)	1
right hemicolectomy	1(4.2)	0(0)	1
Operation time (min)	87.1(28.1)	51.4(20.4)	0.04
Postoperative complications	0(0)	0(0)	1
Postoperative hospital stay (day)	8.1(2.9)	4.3(1.6)	0.04

Group 2a, interval surgery group; Group 2c, surgery group for recurrence of appendicitis.

Data are expressed as mean (SD) or absolute number of patients (%).

## 5 Discussion

An appendiceal mass is the result of a walled-off perforation of the appendix and represents a wide pathological spectrum ranging from an inflammatory mass that consists of the inflamed appendix, some adjacent viscera and the greater omentum to a periappendiceal abscess. Appendiceal mass develops in 2–7% of acute appendicitis and has the trend towards increasing in frequency due to the application of the conservative treatment of the uncomplicated acute appendicitis only with antibiotics (without surgery). Some cases of unsuccessful outcome of antibiotic therapy increases the risk of complicated appendicitis and the development of appendiceal mass [[11], [12], [13]].

The management of the appendiceal mass and abscess remains controversial. In general, there are three methods of treatment: the most widely used, the,classical” conservative management followed by interval surgery, totally conservative management without interval surgery and emergency surgery [1,4,10,14].

The most widely used method of treatment is considered to be the non-operative method by Ochsner in 1901 [15]. Currently this method implicates starting treatment with broad spectrum antibiotics and infusion therapy. In case of improvement in patients' condition, interval surgery is indicated after 6–8 weeks [3,13,16]. In case of existence or formation of appendiceal abscess, US or CT-guided PCD is indicated [1,5,10,17]. If patient's condition is not improving, surgical intervention should be performed. In our study 11 patients out of 47 conservatively treated patients had been performed US or CT-guided PCD. Complications were developed in 2 (18.2%) patients out of 11: intra-abdominal abscess – in one patient and intestinal fistula - in another one. Both patients were operated (ileocecectomy and right hemicolectomy). Several authors mention about the postoperative complications after US or CT-guided PCD [5,18]. Interval surgery is supposed to prevent recurrence of appendicitis. However, nowadays the need of interval surgery after conservative treatment is disputable. The reasons of this controversy are the data indicating the low rate of recurrences of acute appendicitis (about 10%), in the case the conservative treatment of appendiceal mass and abscess is not followed by interval surgery [1,4,6,[19], [20], [21]]. According to our data, recurrence of appendicitis developed only in 3 (13%) out of 23 patients. At the same time an interesting fact was fixed: US or CT PCD were performed in 5 patients out of 23 and 3 of them developed recurrence of appendicitis. This fact points out that using US or CT PCD increases the risk of recurrence of appendicitis. Our research also showed significantly diminished operation time and postoperative hospital stay in the group of patients operated due to the recurrence of appendicitis compared to interval surgery group. Based on the above-mentioned we support the group of the researchers who think that appendiceal mass and abscess may not need interval surgery. At the same time, patients with US or CT PCD have to be considered as of having high risk of recurrence of appendicitis.

Conservative treatment is associated with a risk of missing or delaying hidden pathologies such as malignant disease, Crohn's disease and other diseases [1,3,4,22]. Reference data indicate to increased frequency of colon cancer presented by appendiceal mass: 5.9–12% of the patients with appendiceal mass are diagnosed with colon cancer [23,24]. Thereby conducting the procedures such as colonoscopy and CT are important to be performed for detection of such diseases after conservative treatment. There is no general consensus regarding the right time to perform such investigations. It is believed, that such investigations can be performed safely 4–6 weeks after the acute episode. According to the literature, these investigations, especially colonoscopy, are most important and necessary particularly in patients aged 40 years and over [1,5,14,23,25]. In our research 3 (4.1%) patients out of 74 were not diagnosed with acute appendicitis as a main cause of appendiceal mass: in two patients – it was caecal cancer (60 years old man from the emergency surgery group and 36 years old man from the conservative management group) and in one case – Crohn's disease (30 years old woman from the conservative management group). Due to the fact that from these three patients two were under 40 years of age, we think that all patients regardless their age must undergo CT and colonoscopy on the stage of conservative treatment.

According to the reference data, emergency surgery in the case of appendiceal mass and abscess is not supported by majority of surgeons. The main reasons are: 1. Need of the colon resections (ileocecectomy or right hemicolectomy) instead of appendectomy in 25–30% patients due to the acute inflammation in the right iliac fossa, edema and/or vulnerability of small and large intestines, and adhesions; 2. High frequency of the postoperative complications [1,[3], [4], [5],^17^,^18^,^22^]. Although there is a group of few members of surgeons who opt in the idea of emergency surgery. By their opinion, the superiority of the emergency surgery is avoiding the need of longitudinal follow-up and re-hospitalization due to the elective surgery. This method avoids misdiagnoses and promptly deals with any unexpected ileocecal pathology that masquerades as an appendiceal mass [7,26,27].

Our data shows that colon resection was performed in 25.9% in the emergency surgery group. Statistically significant increase in the timing of surgical operation, more postoperative complications and longer postoperative hospital stay – these are the parameters characteristic for the emergency surgery group compared to the interval surgery group. Following to these results we prefer to use conservative treatment method in case of appendiceal mass and abscess.

The limitations of the study are the non-randomized design, small sample size, exclusion of immunocompromised patients, and patients in ASA 4 and 5 groups. Larger, randomized multicenter studies are needed for further clarification in choosing optimal treatment approach.

## 6 Conclusions

Conservative treatment without interval surgery seems to be the preferred method for treatment of appendiceal mass and abscess. Patients can be operated on only in case of recurrence of appendicitis. US or CT PCD of appendiceal abscess presents the risk-factor for the development of recurrence of appendicitis. CT and colonoscopy within 4–6 weeks after completing the conservative treatment is recommended to be performed in all patients to prevent the pathologies of right iliac fossa (cancer, Crohn's disease).

## Sources of funding

This research did not receive any specific grant from funding agencies in the public, commercial, or non-profit sectors.

## Provenance and peer review

Not commissioned, externally peer reviewed.

## Declaration of competing interest

The authors declare that they have no conflict of interests.
